# Use of rituximab in glomerulopathies

**DOI:** 10.1590/2175-8239-JBN-2022-E004

**Published:** 2022-02-23

**Authors:** Cristiane Bitencourt Dias, Viktoria Woronik

**Affiliations:** 1 Universidade de São Paulo, Faculdade de Medicina, Laboratório de Fisiopatologia Renal, São Paulo, SP, Brasil.

The use of rituximab (RTX) has been extended from the treatment of non-Hodgkin’s lymphomas to autoimmune diseases, and since then some unanswered questions have arisen. One of these is the use of this anti-B lymphocyte drug in minimal change disease (MCD) and focal segmental glomerulosclerosis (FSGS), since these are not antibody-mediated diseases. The pathogeneses of MCD and FSGS are still no clear, but in the case of MCD it is caused by dysregulation of T lymphocytes, whereas in the case of primary FSGS it is related to the action of circulating factors produced by inflammatory cells outside the glomerulus, including T lymphocytes. However, it is known that RTX-induced B lymphocyte depletion could modify T cells and deplete approximately 5% of circulating T lymphocytes expressing CD20^1^. Blockade of B lymphocyte is also thought to have indirect effects, such as kinase and caspase inhibition, and thus effects on cell phosphorylation and apoptosis^1^. Recently, sphingomyelin phosphodiesterase acid-like 3b (SMPDL-3b) in podocytes, which when suppressed facilitates actin remodeling, was found in some cases of FSGS. RTX counteracts the loss of SMPDL-3b, keeping actin intact. Therefore, like corticosteroids, calcineurin inhibitors and mycophenolate, this medication also has a non-immunological action on podocyte remodeling^2^.

Duarte I et al.^3^ present 19 patients with glomerular disease treated with RTX, retrospectively analyzed and with two different protocols of posology. Four of the 19 patients had FSGS, one of whom was considered steroid-resistant, whereas two patients both of whom were not steroid-resistant responded to the use of RTX. Other authors have also published case reports in which the response to RTX varied greatly^4^. KDIGO 2020 recommends the use of RTX in MCD and FSGS as an excellent corticosteroid saver, but its use in corticoid-resistant patients is questioned^5^.

Duarte I et al. also present cases of membranous nephropathies and related ANCA vasculitis in which indication for the use of RTX is already better elaborated. In both of these diseases, the use of RTX was not inferior to well-established immunosuppressive treatments^5^. The superiority of RTX has been proven only in the maintenance treatment of ANCA-related vasculitis. However, in severe forms of these diseases, RTX has not been well studied and the recommendation remains to use corticosteroids and cyclophosphamide (CYC)^5^.

RTX has been tested in class III and IV lupus nephritis (LN) patients and until now its role has not been clearly established. In most studies, RTX was used as add-on therapy in combination with CYC or mycophenolate mofetil and methylprednisolone followed by prednisolone as induction therapy^6^. Different doses and regimes of RTX were tested for LN: in the LUNAR protocol,^6^ 1000 mg RTX was prescribed on days 1, 15, 168, and 182, while other protocols preferred the regimen of four infusions (375mg/m^2^) one week apart. RTX may induce different responses in patients of different ethnic and racial backgrounds. In some studies, Caucasians had a better response than Latinos and African Americans, while in the LUNAR protocol, patients of African descent had a higher renal response rate when RTX was compared to placebo (75% vs 45%, respectively).

Different response criteria based on proteinuria and renal function resulted in different remission rates in the studies. No statistical differences were observed when comparing complete and partial remission rates of RTX groups with those of conventionally treated groups in various protocols.^7^ From and immunological perspective, there is a direct correlation between the clinical renal response to RTX and the onset and duration of B-cell depletion, calling attention to the effective therapeutic dose of RTX and the peripheral null count of CD20/CD19 lymphocytes. Side effects of RTX are mild and mostly related to infusion reactions. Infections and neutropenia are described.

Duarte I et al. used RTX in four LN patients, with complete remission in two, no response in one, and rapid deterioration of renal function in one. Condon et al.,^8^ in a prospective study known as Rituxilup, proposed a protocol that spared corticosteroids by administering two doses of RTX and methylprednisolone two weeks apart without using corticosteroids at follow-up. Their results are impressive, with 52% having complete response and 34% having partial response after 52 weeks. These results allow us to use RTX as a corticosteroid-sparing agent with outstanding benefits for patients.

Dosing and monitoring of RTX has been a challenge. The two main forms of administration are 375 mg/m^2^ intravenously once a week for four weeks or 1 g intravenously on days 1 and 14. Some publications recommend monitoring of the administrations of the new drug with CD19 count or gamma globulin values. However, this is inconclusive and the medication administrations remain in these two fixed doses and their repetition six months after the first administration if the disease has not remitted. Studies with MCD using a single dose of RTX showed that there was CD19 depletion in 83% of patients up to the third month. When CD19 was measured monthly, there was no correlation between CD19 and the return of proteinuria, with a relapse of proteinuria occurring even in patients with depleted CD19. This may be explained by the fact that CD19 only labels serum B lymphocytes, not tissue or memory cells^9,10^.

It is important to remember that RTX also causes infections, and the prophylactic use of trimethoprim-sulfamethoxazole is recommended for the prevention of pneumocystis jirovecii infection. Authors have already associated greater infections with the use of RTX in diabetics, previous use of other immunosuppression drugs, and with an accumulated dose of RTX^11^.
